# Supplementary material for: LongHealth: A Question Answering Benchmark with Long Clinical Documents
Source: J Healthc Inform Res. 2025 Jun 14;9(3):280–96. doi: 10.1007/s41666-025-00204-w (PMC12290132; doi:10.1007/s41666-025-00204-w)
Supplement: Supplementary file 1 — Supplementary file1 (DOCX 29 KB) [file 41666_2025_204_MOESM1_ESM.docx]

**Supplementary Material**

**Table S1:** Overview of baseline models tested on *LongHealth.*

| **Model** | **Parameters** | **Context Length**  **(Tokens)** | **Architecture** | **Access** |
| --- | --- | --- | --- | --- |
| Mistral-Small-24B-Instruct-2501 | 24B | 32k | Transformer | Apache‑2.0 OSS |
| Llama-4-Scout-17B-16E | 17B active / 109B total | 10M | Mixture-of-Experts | Source‑available (Llama 4 Community License) |
| Mixtral-8x7B-Instruct-v0.1 | 45B (8×7B) | 32k | Mixture-of-Experts | Apache‑2.0 OSS |
| gpt-3.5-turbo-1106 | Undisclosed | 16k | Transformer | API-based |
| Yi-34B-200k | 34B | 200k | Transformer | Apache‑2.0 OSS |
| Mistral-7B-Instruct-v0.2 | 7B | 32k | Transformer | Apache‑2.0 OSS |
| vicuna-13b-v1.5-16k | 13B | 16k | Transformer | Apache‑2.0 OSS |
| zephyr-7b-beta-16k | 7B | 16k | Transformer | MIT OSS |
| longchat-13b-16k | 13B | 16k | Transformer | Apache‑2.0 OSS |
| vicuna-7b-v1.5-16k | 7B | 16k | Transformer | Apache‑2.0 OSS |
| longchat-7b-v1.5-32k | 7B | 32k | Transformer | Apache‑2.0 OSS |
| Yi-6B-200K | 6B | 200k | Transformer | Apache‑2.0 OSS |

**Table S2:** Overview of costs for different LLMs per 1,000,000 tokens.

| **Model** | **Costs per 1,000,000 tokens** |
| --- | --- |
| gpt-3.5-turbo-1106* | $0.50 |
| gpt-4-0613* | $30 |
| Llama-4-Scout-17B-16E** | $0.27 |
| Llama-3.3-70B-Instruct** | $0.88 |
| Mistral-Small-24B-Instruct-2501** | $0.80 |

*OpenAI API pricing, accessed Feb. 28th, 2025

**togetherAI API pricing, accessed Feb. 28th, 2025

**Table S3:** Overview of costs for different LLMs on Task 1.

| **Model** | **Cost/Run (16,000 tokens context length)** | **Cost/5 Runs (16,000 tokens context length)** | **Cost Task 1 (5 runs, context length from 4,000 to 32,000, increasing by 4,000 each step)** |
| --- | --- | --- | --- |
| gpt-3.5-turbo-1106 | $3.2 | $16.02 | $141.92 |
| gpt-4-0613 | $378.45 | $961.22 | $8515.23 |
| Llama-4-Scout | $1.73 | $8.63 | $76.44 |
| Llama-3.3-70B | $5.63 | $28.16 | $249.46 |
| Mistral-Small-24B-Instruct-2501 | $5.12 | $25.59 | $115.13 |

**Table S4:** Overview of costs for different LLMs on Tasks 2 and 3.

| **Model** | **Cost/Run (16,000 tokens context length)** | **Cost/5 Runs (16,000 tokens context length)** | **Costs Task 2 & 3 (nearly identical texts and questions)** |
| --- | --- | --- | --- |
| gpt-3.5-turbo-1106 | $3.23 | $16.13 | $32.27 |
| gpt-4-0613 | $193.59 | $967.96 | $1935.92 |
| Llama-4-Scout | $1.74 | $8.7 | $17.39 |
| Llama-3.3-70B | $5.67 | $28.36 | $56.72 |
| Mistral-Small-24B-Instruct-2501 | $5.15 | $25.76 | $51.52 |
